# Characteristics of soil microbial communities in farmland with different comprehensive fertility levels in the Panxi area, Sichuan, China

**DOI:** 10.3389/fmicb.2023.1237409

**Published:** 2023-09-14

**Authors:** Yadong Xu, Taibo Liang, Huaxin Dai, Zhen Zhai, Yulan Chen, Guangting Yin, Yanling Zhang, Caipeng Yue

**Affiliations:** ^1^School of Agricultural Sciences, Zhengzhou University, Zhengzhou, Henan, China; ^3^Zhengzhou Tobacco Research Institute of CNTC, Zhengzhou, Henan, China; ^2^Henan Funiu Mountain Biological and Ecological Environment Observatory Research Project, Zhengzhou, Henan, China; ^4^Liangshan Branch of Sichuan Tobacco Company, Xichang, Sichuan, China; ^5^China Tobacco Henan Industrial Co., Ltd, Zhengzhou, Henan, China

**Keywords:** comprehensive fertility, soil microorganism, environmental factors, community assembly, neutral theory

## Abstract

Soil bacterial communities are intricately linked to ecosystem functioning, and understanding how communities assemble in response to environmental change is ecologically significant. Little is known about the assembly processes of bacteria communities across agro-ecosystems, particularly with regard to their environmental adaptation. To gain further insights into the microbial community characteristics of agro-ecosystems soil in the Panxi area of Sichuan Province and explore the key environmental factors driving the assembly process of the microbial community, this study conducted field sampling in major farmland areas of Panxi area and used Illumina MiSeq high-throughput sequencing technology to conduct bacterial sequencing. Soil organic matter (SOM), alkali-hydrolyzed nitrogen (AN), available phosphorus (AP), available potassium (AK) and other environmental factors were determined. The membership function method and principal component analysis method were used to evaluate the fertility of the soil. The results revealed minimal differences in alpha diversity index among samples with different comprehensive fertility indices, while NMDS analysis showed that community differences between species were mainly reflected in high fertility and low fertility (R: 0.068, p: 0.011). Proteobacteria, Acidobacteria and Actinobacteria were the main types of microbial communities, accounting for more than 60% of the relative abundance. Proteobacteria accounted for a higher proportion in the high fertility samples, while Acidobacteria and Actinobacteria accounted for a higher proportion in the middle and low fertility samples. Both the neutral theoretical model and zero model analysis showed that the microbial communities in tobacco-planting soil with different comprehensive fertility indices presented a random assembly process. With the increase in environmental distance difference, the diversity of the microbial community in medium and low-fertility soil also increased, but there was no significant change in high-fertility soil. Redundancy analysis showed that pH and SOM were the key factors affecting microbial community composition. The results of this study can provide a theoretical reference for the study of environmental factors and microbial communities in tobacco-growing soil.

## 1. Introduction

Tobacco (*Nicotiana tobacum* L.) is a special leaf cash crop that is widely planted worldwide (Deng-Shan et al., 2019; Yan et al., 2020). Appropriate soil fertility is an important basis for tobacco’s high quality and suitable yield. The abundance and shortage of soil nutrients and the intensity of supply directly affect the growth and development, yield and quality of tobacco (Zhang J. et al., 2016). The degree of coordination among nutrients is an important factor affecting the ability of tobacco plants to absorb nutrients. Soil microorganisms are the catalyst of material circulation and energy flow between plants and the soil ecosystem, and are an important part of soil quality (Saleem et al., 2019). Soil physical and chemical properties have important effects on soil microbial community diversity, community structure and assembly process (Xu et al., 2019, 2020; Jiao et al., 2021). Although there are many studies on soil microorganisms and soil physical and chemical properties in a single habitat, there is a lack of comprehensive evaluation of soil fertility in a large scale, especially focusing on the characteristics of microbial communities under integrated soil fertility.

Integrated soil fertility is a comprehensive manifestation of the complex interactions among the chemical, biological and physical components of the soil system (Yujian and Xueqin, 2012). The level of integrated soil fertility affects the potential production of crops. Soil integrated fertility is mainly reflected in the characteristics of organic matter, macroelements and essential microelements (Santos et al., 2021). There is no unified standard for the comprehensive evaluation method of soil fertility in the world. Different comprehensive evaluation methods of soil fertility are often selected according to the actual local situation. In recent years, researchers have applied multivariate statistical analysis, cluster analysis, factor analysis, principal component analysis (PCA) and fuzzy mathematics to the comprehensive evaluation of soil fertility, and obtained comprehensive indicators reflecting the level of soil fertility by processing a large amount of information (Luo et al., 2004; Yujian and Xueqin, 2012; Wu et al., 2018).

Soil comprehensive fertility is a dynamic development process and changes with land use time. By using principal component analysis and fuzzy mathematics, Li et al. (2022) found that after 20 years of continuous use, the soil comprehensive fertility of tobacco planting in Nanyang was significantly improved. Different soil types have different soil fertility due to different climate environment. Wang et al. (2014) used fuzzy comprehensive evaluation method to evaluate the comprehensive fertility of dry red soil, red soil, brown soil, yellow brown soil, purple soil, paddy soil and limestone soil in Huili County. The results showed that the comprehensive fertility indices of the seven soil types in Huili tobacco area were: limestone soil > yellow brown soil > paddy soil > red soil > brown soil > purple soil > dry red soil. Most studies only focused on the soil nutrient status in different areas or different comprehensive fertility indices, and few studies analyzed the characteristics of microbial communities and key environmental factors.

Panxi area is located in the southwest of Sichuan Province, which is one of the main producing areas of high-quality flue-cured tobacco. Understanding the comprehensive fertility and microbial community characteristics of tobacco-growing soil in this area has important guiding significance for guiding rational fertilization of tobacco fields and improving tobacco quality. Thus, based on the comprehensive fertility evaluation of tobacco-growing soil in Panxi area, we focused on exploring the characteristics of soil microbial community and exploring the synergistic relationship between microorganisms and soil nutrients under different comprehensive fertility conditions, and we hypothesized that (1) the soil microbial community composition and diversity were different under different comprehensive fertility indices and (2) the key influencing factors of soil microbial community were also different.

## 2. Materials and methods

### 2.1. Study area

Panxi region is located in the southern part of Sichuan Province (26°3′~29°27′ N, 100°15′~103°53′ E), and administrable includes Liangshan Yi Autonomous Prefecture and Panzhihua City. This region is a subtropical monsoon climate zone with distinct dry and wet, abundant solar and thermal resources, abundant rainfall, large daily temperature difference and small annual temperature difference. With unique ecological conditions of high-quality flue-cured tobacco planting, it is one of the country’s high-quality flue-cured tobacco production areas. The study area has complex and diverse landforms, high in the northwest and low in the southeast, with interleaved mountains, deep valleys, plains, basins and hills. The total area of tobacco planting in the 13 main tobacco-producing counties in the Panxi region is 75,300 hectares, which is mainly distributed between 1,000 and 2,500 m above sea level, and the proportion of tobacco planting accounts for more than 65% of the cultivated land area in this region (Zhang et al., 2013).

### 2.2. Soil sampling

To avoid the influence of fertilization on the representativeness and evenness of sampling, sampling was conducted after tobacco leaf harvest. A total of 123 representative tobacco fields were selected, and a 5-point sampling method was used to collect topsoil (10 cm in depth, 5 cm in diameter) in each tobacco field. For the specific sampling point information, see Supplementary Table S1. These samples were sieved with 2 mm mesh to remove the visible plant roots, rocks, litter and debris. A portion of each soil sample was immediately shipped from the field to the laboratory in an ice box and immediately stored at −80°C for DNA extraction. The remaining portion of soil samples was air-dried at room temperature and stored for physicochemical analysis.

### 2.3. Determination of soil abiotic properties

Soil abiotic properties factors, including soil organic matter (SOM), pH, total nitrogen (TN), available nitrogen (AN), available phosphorus (AP), available potassium (AK) were measured as previously described (Ding et al., 2015). Water soluble chlorine (WSCl) was determined by silver nitrate titration, water soluble calcium (WSCa) and magnesium (WSMg) were determined by EDTA titration, and available copper (ACu), zinc (AZn), iron (AFe) and manganese (AMn) were determined by hydrochloric acid extraction and atomic absorption spectrophotometry (Bao, 2000).

### 2.4. Soil DNA extraction, PCR amplification, and sequencing of the 16S rRNA gene

The extraction of soil DNA was conducted from each soil sample using a PowerSoil DNA Isolation Kit (MoBio Company, United States) following the manufacturer’s instructions. The total genomic DNA was extracted from 1.5 g of fresh soil (0.5 g × 3) and then mixed. The NanoDrop (ND-2000, NanoDrop Technologies, Wilmington, United States) was employed to quantify and assess the quality of DNA samples. Using a 2% agarose gel electrophoresis to confirm the integrity of the DNA extracts and storing all extracted DNA from soil in −80°C refrigerator (Zhang X. et al., 2016). Using PCR technology to bacterial 16S rRNA gene amplification, V4~V5 region of universal primers to 515F (5′-GTGCCAGCMGCCGCGGTAA-3′)/926R (5′-CCGTCAATTCMTTTGAGTTT-3′; Wang et al., 2022). The reaction system and conditions of twice PCR amplification are described in Chen R. et al. (2021). The amplified products was detected by 2% agarose gel electrophoresis and recovered by a QIAamp DNA Micro Kit (Qiagen, Valencia, CA, United States), quantified by FTC-3000TM real-time PCR instrument, and the library was constructed after homogenization and mixing. Finally, paired-end sequencing of the amplicon library was performed on an Illumina MiSeq platform from TinyGene Bio-Tech Co., Ltd. (Shanghai, China).

### 2.5. High-throughput sequencing data processing

The QIIME (Quantitative Insights into Microbial Ecology, v1.8.0) workflow was employed to identify the interrogative sequence. The UCHIME (v5.2.236) algorithm was used to filter raw flowgrams and remove noise and chimeras (Edgar et al., 2011). Complete Linkage clustering of the remaining sequences using the UCLUST method (Edgar, 2010) and operational units (OTUs) were classified using a 97% nucleotide sequence similarity cutoff. Based on taxonomic information, statistical analysis of community structure was carried out at the phylum levels. Community Alpha diversity indices were performed using MOTHUR soft (Version 1.33.3).

### 2.6. Statistical analyses

One-way ANOVAs were performed using SPSS version 20.0 (SPSS Inc., Chicago, United States) to examine differences between means. In this case, the least significant difference (LSD; for each sampling instance; *p* < 0.05) was used to separate the means between different treatments within respective sampling instances. Correlations between the soil bacteria species compositions and soil properties were determined using redundancy analysis (RDA) with CANOCO version 5.0. Pearson correlations and Mantel test were visualized using R software via the “linkET” package (version 0.0.2.4; Huang, 2021).

### 2.7. Network analysis and assembly processes for bacteria community

To determine the complex ecological interactions among the microorganisms, network analysis was performed using the open network analysis pipeline (Molecular Ecological Network Analyses Pipeline, http://ieg2.ou.edu/MENA/main.cgi). Hub and connector taxa were determined by among-module connectivity (Pi) and within-module connectivity (Zi), with nodes categorized into module hubs (Pi < 0.62 and Zi > 2.5), connectors (Pi > 0.62 and Zi < 2.5), network hubs (Pi > 0.62 and Zi > 2.5) and peripheral nodes (Pi < 0.62 and Zi < 2.5), as proposed previously (Deng et al., 2012). The neutral community model (NCM) was used to predict the relationship between OTU detection frequency and their relative abundance across the wider metacommunity, to determine the potential importance of stochastic processes on community assembly (Sloan et al., 2006; Chen et al., 2019). We also used a null model analysis to evaluate the assembly processes of communities (Stegen et al., 2013; Dini-Andreote et al., 2015), to classify community pairs into underlying drivers of deterministic processes (e.g., homogeneous selection and variable selection) or stochastic processes (e.g., dispersal limitation, homogeneous dispersal, and “non-dominant”).

### 2.8. Sequence accession numbers

The 16S rRNA gene sequences obtained in our study have been deposited in the NCBI Sequence Read Archive database and the accession number is PRJNA976908.

### 2.9. Integrated fertility index

Soil comprehensive fertility index is a comprehensive index of soil fertility. Firstly, the membership value of each index was calculated according to the effect curve of soil fertility index (SOM, pH, TN, AN, AP, and AK) on crops. The weight of each index was calculated by the principal component analysis method, and then the comprehensive evaluation index value of soil fertility was calculated according to the additive and multiplication principle of fuzzy mathematics, with the following equations (Xiao et al., 2022):

(1) $$IFI=\overset{n}{\underset{i=1}{\sum }}W_{i}N_{i}$$

where Wi and Ni represent the membership value and weight value of the ith soil fertility factor, respectively. The common membership function can be divided into S-type and Parabola-type functions. For the convenience of calculation, the curve function is converted into a broken line function, and the corresponding function expression and broken line graph are shown in Equations 2, 3 (Xiao et al., 2022).

S-type function:


(2) $$f\left(x\right)=\{\begin{array}{l} 1.0\\ 0.1+0.9\left(x-L\right)/\left(U-L\right)\\ 0.1 \end{array}\begin{array}{l} x\geq U\\ L<x<U\\ x\leq L \end{array}$$

Parabola-type function:


(3) $$f\left(x\right)=\{\begin{array}{l} 0.1\\ 0.1+0.9\left(x-L\right)/\left(O_{1}-L\right)\\ 1.0\\ 1.0-0.9\left(x-O_{2}\right)/\left(U-O_{2}\right) \end{array}\begin{array}{l} x\leq L,x\geq U\\ L<x<O_{1}\\ O_{1}\leq x\leq O_{2}\\ O_{2}<x<U \end{array}$$

The membership function types of each factor and the specific values represented by L, O_1_, O_2_, and U are detailed in Table 1. Based on the above calculation results, we obtained the comprehensive fertility index of all sample sites, ranging from 0.1038 to 0.9885. According to previous relevant studies (Wang et al., 2015; Song et al., 2016; Fan et al., 2021), we comprehensively considered the value range of the comprehensive fertility index and defined the low fertility index (LF) as less than 0.55, the medium fertility index (MF) between 0.55 and 0.75, and the high fertility index (HF) as greater than 0.75. See Table 2 for the specifically related properties.

**Table 1:** Types of membership function and limitation value.

Types of membership function	Parabola-type function				S-type function	
	pH	SOM	AN	TN	AP	AK
L	5	15	30	0.9	20	100
O_1_	5.5	25	50	1.4		
O_2_	7	35	70	2.5		
U	7.5	45	100	3.5	40	150

L, Lower limited value; O_1_, Optimal value of lower limitation; O_2_, Optimal value of upper limitation; U, Upper limitation value.

**Table 2:** Soil properties of LF, MF, and HF.

	LF	MF	HF
SOM (g/kg)	22.9 ± 2.24a	21.82 ± 0.96a	23.97 ± 0.82a
pH	6.19 ± 0.17b	6.43 ± 0.17ab	6.82 ± 0.16a
TN (g/kg)	1.29 ± 0.12a	1.23 ± 0.04a	1.25 ± 0.04a
AN (mg/kg)	85.57 ± 9.09a	81.09 ± 3.74a	75.26 ± 1.75a
AP (mg/kg)	28.59 ± 4.13b	36.06 ± 3.86b	57.59 ± 4.85a
AK (mg/kg)	206.54 ± 16.49b	272.59 ± 20.31a	303.85 ± 25.04a
WSCa (mg/kg)	66.26 ± 9.53b	102.46 ± 10.2a	78.24 ± 9.84ab
WSMg (mg/kg)	21.63 ± 2.57a	24.16 ± 1.91a	25.97 ± 3.12a
ACu (mg/kg)	2.26 ± 0.4a	1.62 ± 0.18a	1.62 ± 0.14a
AZn (mg/kg)	1.98 ± 0.24a	1.95 ± 0.23a	2.39 ± 0.33a
AFe (mg/kg)	65.14 ± 16.11b	37.41 ± 4.58b	30.45 ± 3.07a
AMn (mg/kg)	36.12 ± 4.81a	42.5 ± 4.78a	32.1 ± 3.67a
ACl (cm/kg)	0.15 ± 0.01a	0.13 ± 0.01a	0.15 ± 0.04a

Different lowercase letters represent a significant difference among soils with different comprehensive fertility levels (*p* < 0.05).

## 3. Results

### 3.1. Soil bacteria diversity and community composition

**Figure 1:**
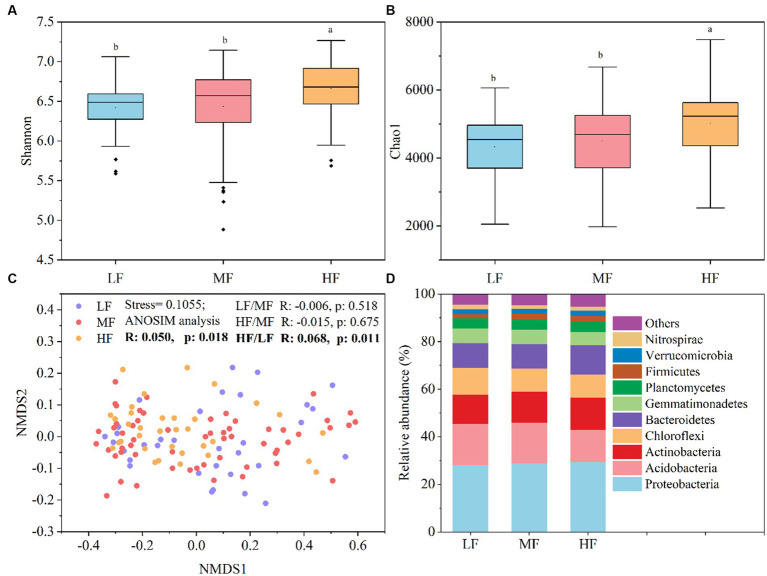
Soil bacteria diversity and community composition. **(A)** Shannon index. **(B)** Chao1 index. **(C)** NMDS showed the structure of bacteria community. **(D)** Relative abundance of community composition at the phylum level. Lowercase letters indicate significant differences between soils with varying comprehensive fertility levels at a significance level of 0.05 in panels **(A,B)**. We define species with an average relative abundance below 1% in all samples as “Others” in panel **(D)**.

Figure 1 showed that the index of Shannon (Figure 1A) and Chao1 (Figure 1B) in HF were significantly higher than that in MF and LF. The NMDS and ANOSIM analysis indicate that there were significant differences among the three groups of the bacteria community structure (R > 0, *p* < 0.05), and the major differences were observed between LF and HF (Figure 1C). The HF soils harbored higher relative abundance of Proteobacteria, Actinobacteria, Bacteroidetes, and Firmicutes, while the LF soils harbored higher relative abundance of Acidobacteria and Chloroflexi (Figure 1D).

### 3.2. Effects of soil properties on bacterial communities

**Figure 2:**
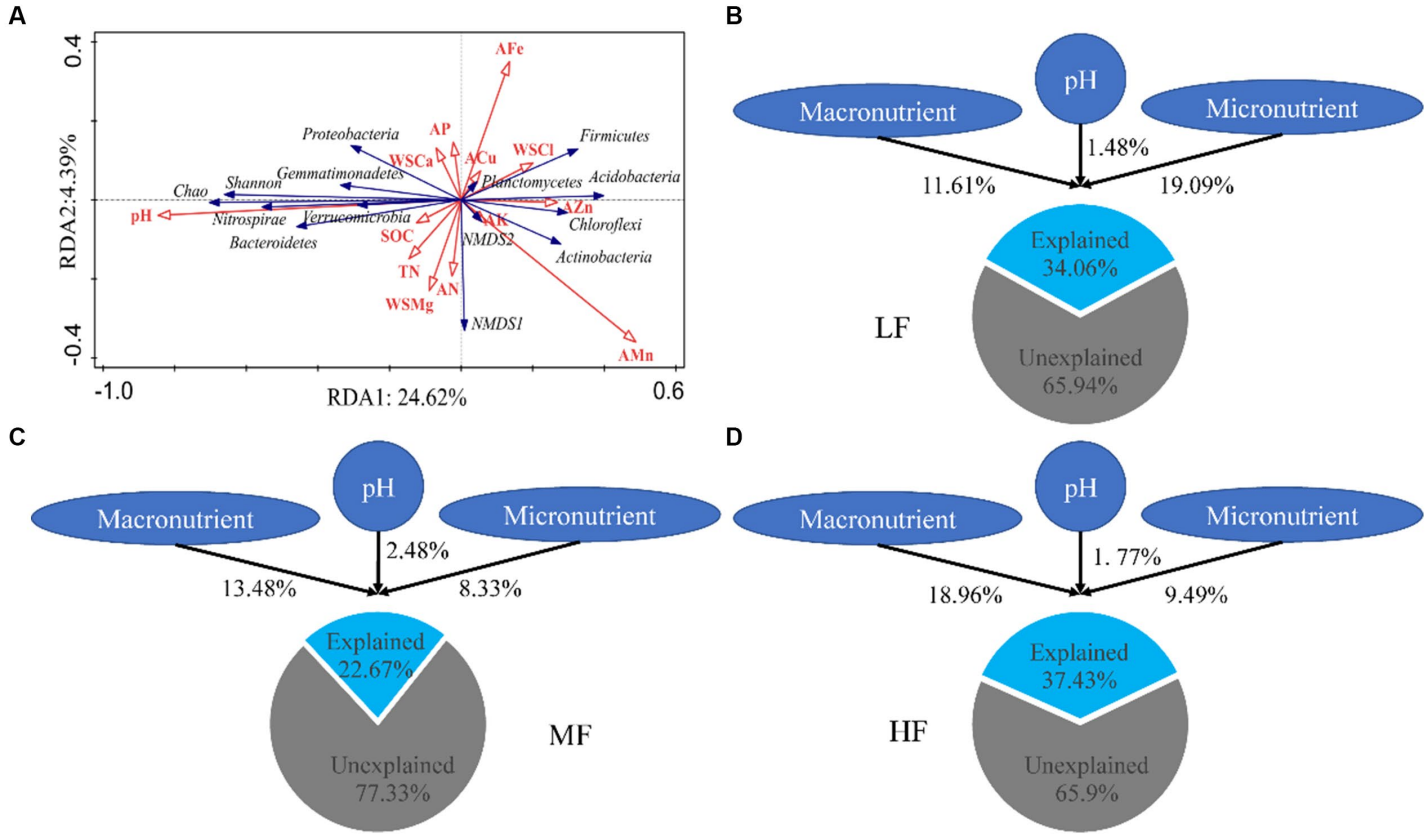
Redundancy analysis of soil properties on bacterial diversity and communities **(A)**. Variance partition analysis of soil properties on microbial communities in LF **(B)**, MF **(C),** and HF **(D)**. Macronutrient encompass SOM, TN, AN, AP, AK, WSCa, WSMg; Micronutrient encompass WSCl, ACu, AZn, AFe, AMn.

The results of RDA showed that pH, SOM, and WSCa may be the key factors which had a positive correlation with Proteobacteria, Bacteroidetes, Shannon index and Chao index and had a negative correlation with Acidobacteria (Figure 2A; Table 3). We also categorized soil properties into pH, macronutrient, and micronutrient, and quantified their contributions to soil bacterial OTUs changes using variance partitioning analysis (VPA; Figures 2B–D). The results showed that soil properties explained 34.06%, 22.67%, and 37.43% of variation in LF, MF, and HF, respectively. Many more variations could not be explained, ranging from 62.57% to 77.33%. The explanation of macronutrient factor for variation showed a gradual increase trend, which ranged from 11.61% to 18.96%, and the explanation of micronutrient factor was mainly reflected in LF.

**Table 3:** Conditional term effects of RDA.

Name	Explains %	Pseudo-F	*p*
pH	**17.8**	**26.6**	**0.002**
SOM	**2.6**	**3.9**	**0.018**
WSCa	**1.8**	**2.8**	**0.04**
AMn	1.2	1.9	0.14
AFe	1.3	2	0.14
TN	2.1	3.3	0.032
AN	1.2	1.9	0.134
WSMg	0.6	1.1	0.378
AZn	0.7	1.1	0.314
AP	1.1	1.9	0.14
WSCl	<0.1	0.2	0.95
AK	<0.1	0.1	0.978
ACu	<0.1	<0.1	0.992

The bold values represent significant (*p* < 0.05) variables.

### 3.3. Co-occurrence network structure and the keystones

**Figure 3:**
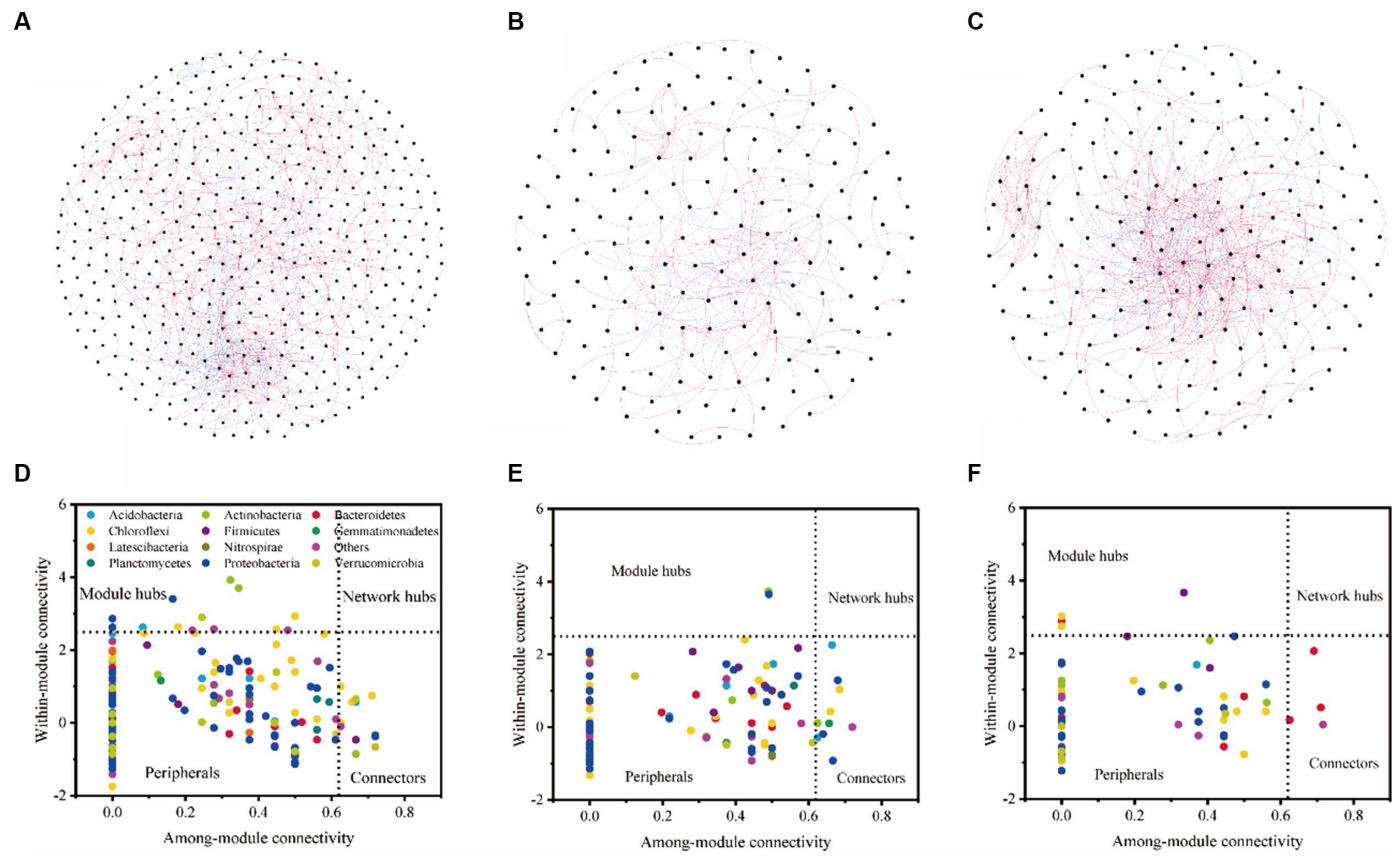
Bacterial co-occurrence networks and the Z-P plot in LF **(A,D)**, MF **(B,E)**, and HF **(C,F)**. Nodes represent individual OTUs; red edges represent significant positive correlations and light blue edges represent significant negative correlations (ρ > 0.6, *p* < 0.001). For detailed network properties, see Table 3.

We found that with the same similarity thresholds, the network had different properties (Figures 3A–C; Table 4). There were more nodes and edges in LF and the least in HF. The average degree was higher and the average path distance was lower in MF. In HF, the proportion of positive correlations was the highest, and there was little difference between MF and LF. We identified a series of module hubs and connectors hubs based on their within-module connectivity (Zi) and among-module connectivity (Pi), which could be regarded as keystones that play key roles in shaping network structure. The number of module hubs and connectors hubs were higher in LF and MF groups than that in HF group. The keystones mainly belonged to Actinobacteria, Proteobacteria, Acidobacteria, Chloroflexi, Verrucomicrobia, Firmicutes, and Gemmatimonadetes in LF, belonged to Actinobacteria, Proteobacteria, Acidobacteria, Chloroflexi, and Gemmatimonadetes in MF, and belonged to Firmicutes, Bacteroidetes, Chloroflexi in HF (Figures 3D–F; Table 4).

**Table 4:** The properties of co-occurrence networks.

Empirical network indexes	LF	MF	HF
Nodes	457	199	169
Links	820	496	243
Average degree	3.59	4.98	2.88
Average path distance	5.39	3.17	4.27
Module	37	25	27
Modularity	0.696	0.516	0.707
Positive correlations (%)	66.59	65.02	73.79
Negative correlations (%)	33.41	34.98	26.21
Peripheral species	430 (94.09%)	187 (93.97%)	161 (95.27%)
Module hubs	14 (3.06%)	2 (1.01%)	4 (2.37%)
Connector hubs	13 (2.84%)	10 (5.03%)	4 (2.37%)
Network hubs	0	0	0

### 3.4. Correlations of the key factors to keystones in each group

**Figure 4:**
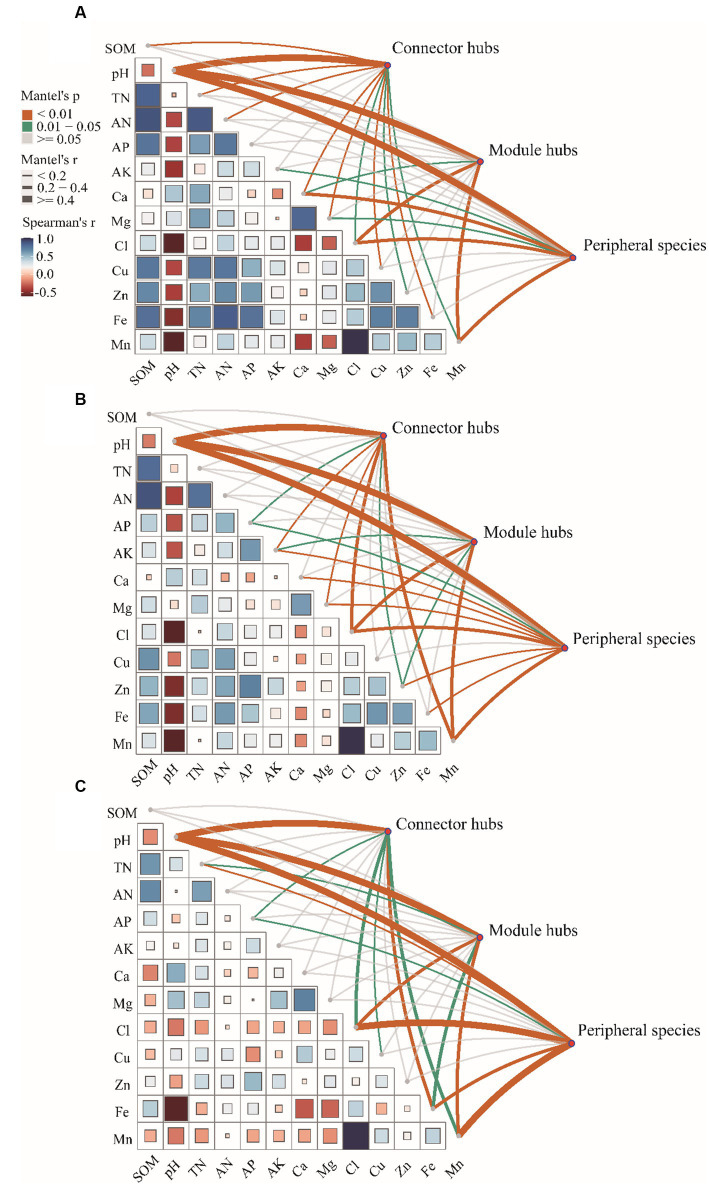
Correlations of the key factors to keystones in each group (**A**: LF, **B**: MF, **C**: HF).

Pairwise comparisons of environmental factors are shown that pH was negatively correlated with other factors except WSCa and WSMg (LF and HF groups; Figures 4A,C) and calcium (MF group; Figure 4B). In LF and MF groups, SOM was negatively correlated with WSCa and pH, but positively correlated with other factors. In HF group, SOM was positively correlated with TN, AN, AP and AFe, but negatively correlated with other factors. We also found that there were more positive correlations among the soil factors in LF and MF groups, which more negative correlations in HF. WSCl and Mn showed a highly significant positive correlation regardless of the group. The mantel test showed that soil properties had significant correlations with the keystones. We observed that pH showed highly significant correlations (*p* < 0.01) with connectors hubs, module hubs and peripheral species in all groups. In LF group, SOM, pH, TN, AN, WSCa, WSMg, WSCl, ACu, AZn, AFe, Mn had significant correlations with the keystones, in MF were pH, AP, AK, WSMg, WSCl, AZn, AMn, and in HF were pH, TN, AP, WSCl, ACu, AFe, AMn.

### 3.5. Soil bacteria community assembly process

**Figure 5:**
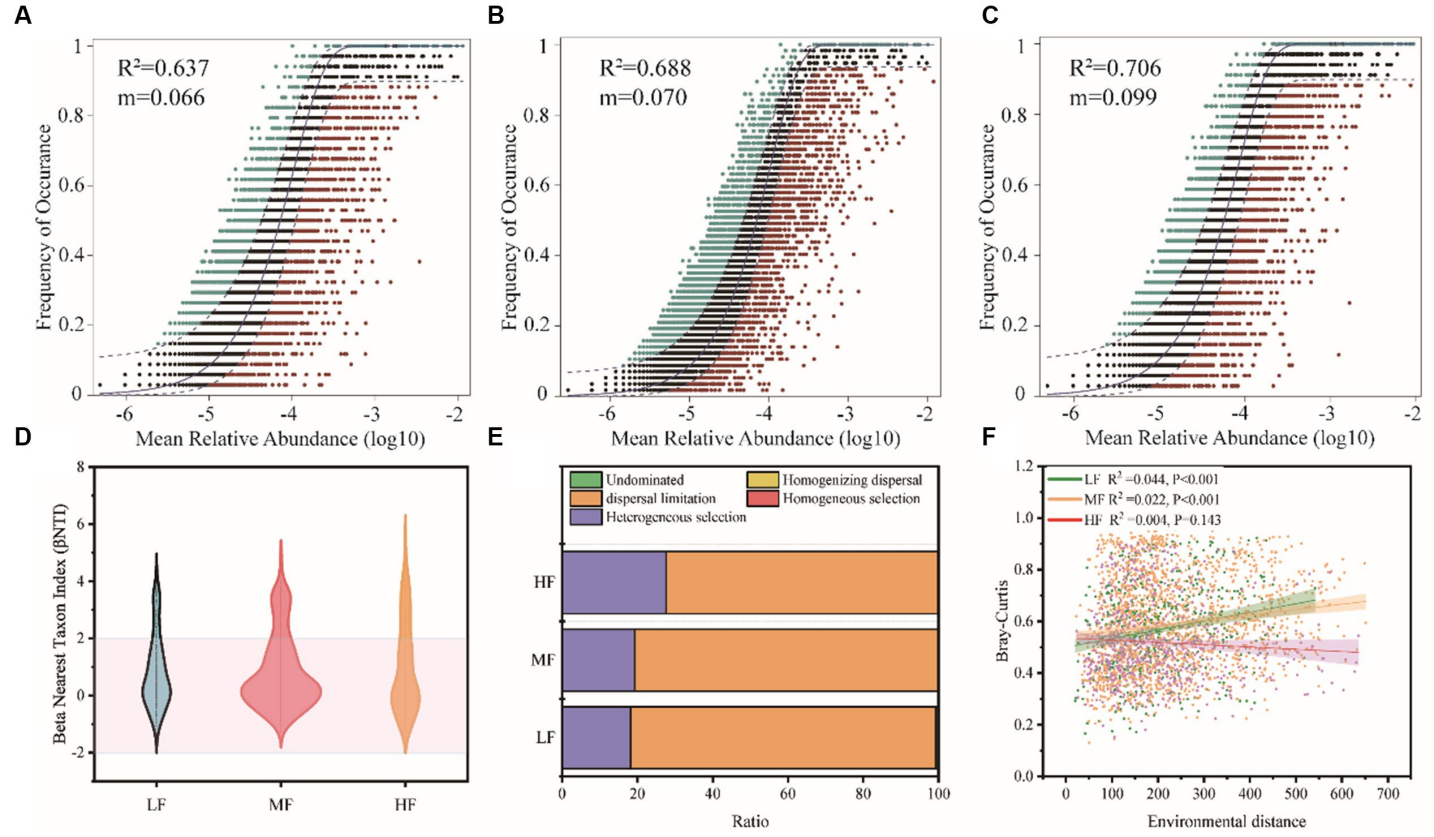
Soil bacteria community assembly process of the different groups. Fit of Sloan’s neutral model for analysis of community assembly processes (OTUs are represented by solid dots) **(A–C)**. Null model analysis of the community assembly processes **(D,E)**. The relationship between environmental distance and microbial community dissimilarity (Bray-Curtis) **(F)**.

The neutral community model (NCM) successfully estimated a large fraction of the relationship between the occurrence frequency of OTUs and their relative abundance variations (Figures 5A–C), with 64.3%, 64.2%, and 74.2% of explained community variance for LF, MF, and HF, respectively. The m value was estimated to be 0.079, 0.062, and 0.084 in LF, MF, and HF, respectively. We also used the null model to calculate betaNTI and Raup-Crick, which found that dispersal limitation and heterogeneous selection were the main ecology process (Figures 5D,E). In LF, MF, and HF, the proportion occupied by dispersal limitation is 81.1%, 80.7%, and 72.2%, the heterogeneous selection is 18.2%, 19.2%, and 27.6%, respectively. We further constructed the relationship between environmental distance and microbial community dissimilarity (Bray-Curtis), and found a significant increase in bacterial dissimilarity with increasing environmental distance in LF and MF groups, but did not change significantly in the HF group (Figure 5F).

## 4. Discussion

In this study, we have demonstrated that an increase in soil comprehensive fertility index is positively correlated with microbial alpha diversity. Furthermore, our results indicate that the Shannon and Chao1 indices were significantly higher in HF soils compared to MF and LF soils. These findings suggest that enhancing soil fertility can provide a more nutrient-rich environment for microbes, leading to increased diversity (Jin et al., 2014; Hartmann and Six, 2023). The difference in beta diversity was mainly between HF and LF, which indicated that microbial community was significantly different between HF and LF. These findings were similar to those of Delgado-Baquerizo et al. (2017), where in the diversity. Regardless of the soil type, Proteobacteria, Acidobacteria, Actinobacteria, Choroflexi, and Bacteroidetes were the richest phylum which was commonly consistent with previous findings that soils usually including some common and ubiquitous bacterial. These results confirm that the major groups in the HF soils are fast-growing bacteria, especially the phyla of Proteobacteria and Bacteroidetes which considered to be eutrophic microbiota (Chen Y. et al., 2021; Li et al., 2021). The phyla of Acidobacteria which considered to be oligotrophic microbiota was the major group in the LF due to their higher substrate affinities (Fierer et al., 2007; Vergin et al., 2013; Xue et al., 2020). Therefore, different comprehensive fertility statuses can affect the heterotrophic strategies of microorganisms. To verify the effects of environmental factors on microbial diversity and community composition, we analyzed the correlation between environmental factors and different microbial properties with the methods of RDA and VPA. The results showed that there was positive correlation or negative correlation between soil environmental factors and some microbial properties. The pH, SOM, and WSCa may be the key factors which had a positive correlation with Proteobacteria, Bacteroidetes, Shannon index and Chao index and had a negative correlation with Acidobacteria. Calcium is a necessary element for plant growth and may directly increase pathogen resistance in tobacco (Niu et al., 2017), therefore, the soil with high fertility index may have more abundant WSCa. It was known that pH and SOM are the key factors that affect soil bacterial community composition and diversity. The range of soil pH considered suitable for tobacco growth is generally 5.5 to 7.5, our study suggests a narrower range of pH that might be suitable for microbial growth. Under the condition of high comprehensive fertility, appropriate pH and organic matter can further promote the proliferation of eutrophic bacteria and increase alpha diversity. Most of the Acidobacteria are acidophilic, whose dynamics could be driven by environmental factors such as pH and some nutrients (Mao et al., 2012; Li et al., 2021), and when pH is close to neutral, there is a negative correlation.

The results of VPA indicate that there are numerous variations that cannot be accounted for by soil properties alone. Notably, the explanation rate of microelements was found to be dominant in LF soil, while that of macroelements was dominant in HF soil. This was mainly due to the fact that the important role of microelements is more obvious due to the imbalance of soil nutrients when the integrated fertility is low, and low soil fertility will further lead to increased plant demand for microelements. This also indicates that in addition to the influence of soil properties on microbial communities, microbial communities themselves also have interactions (Habiyaremye et al., 2021). Co-occurrence network analysis measures the interactions between different microbial taxa by correlation of their abundances across multiple soil samples, and extracts simple patterns from complex interactions to identify cooperative (niche overlap, cooperative exchange or access to resources) or competitive (niche separation, competition for space or resources) relationships between species (Barberán et al., 2012). The positive connections of bacterial networks in soil with different fertility indices were more than negative connections, indicating that bacteria in tobacco soil were more inclined to co-exist in a synergistic and interactive way, and the synergistic effect among bacteria in HF soil was the strongest, meanwhile, compared with LF, they have shorter average path distance in HF and MF, which further confirmed the strong stability of its network structure and function. The Zi-Pi scatter plot showed that the number of module hubs and connectors hubs was higher in LF and MF groups than that in the HF group. The keystones mainly belonged to Actinobacteria, Proteobacteria, Acidobacteria, Chloroflexi, Verrucomicrobia, Firmicutes, and Gemmatimonadetes in LF, belonged to Actinobacteria, Proteobacteria, Acidobacteria, Chloroflexi, and Gemmatimonadetes in MF, and belonged to Firmicutes, Bacteroidetes, Chloroflexi in HF. The mantel test showed that with the increase of comprehensive fertility level, the effect of major factors may be stronger, which will further reduce the significant number of contributing factors. We also found that the pH, Fe and Mn were significantly correlated in all groups. Fe and Mn were trace elements needed for the growth of plants and microorganisms. Lack or excess of Fe and Mn in soil will have adverse effects on plants, resulting in crop health hazards and food safety problems (Welch, 1995; Vatansever et al., 2017). The formation of core microorganisms is not entirely dependent on their relative abundance, but may also be affected by other microbial factors and environmental factors in the network (Shi et al., 2020). Some key species in low abundance flora play more important roles in maintaining material circulation and resisting disturbance than some high abundance flora. This finding is consistent with the results of Mary et al. (1996), Zhang et al. (2018), and Lyons and Schwartz (2001), which confirmed that some low-abundance microorganisms play an active role in driving the material circulation and functional composition of the community and more likely to interact with other microbial community.

The mechanism of community assembly and evolution is further inferred. Our results clearly support the prominent role of stochastic processes in shaping the assembly of soil bacterial biomes. The neutral community model (NCM) is a neutral-based process model, which is a valid approach for inferring stochastic processes acting on community assembly, and has been successfully applied to a wide range of ecological phenomena. This model allows researchers to quantify the importance of processes which are difficult to observe directly but can have large influence on microbial communities (i.e., dispersal and ecological drift; Chen et al., 2019). The value of NCM parameter R^2^ was slightly higher in the HF than LF soils, and according to the calculated Nm values, bacteria dispersal between the sampling sites in HF is likely higher than LF counterparts (Figure 4), indicating that the influence of stochastic processes was stronger in the HF soils. These results indicated that stochastic processes were very important in shaping the bacteria community assembly in all soils. Several key observations also revealed similar results to our finding (Zhang X. et al., 2016). Further, regarding the community immigration rate, the m values in HF were higher compared with LF (Figures 5A,C), indicating the dispersal ability of most bacteria taxa in HF was higher than LF counterparts. We also found the weak decay of community similarity with environment distance in soils, especially in HF. These results might be attributed to the higher habitat homogeneity, soil permeability and connectivity in HF compared with other soils. High dispersal rate can partly overwhelm both environmental selections and ecological drift. Otherwise, the null model analysis showed that stochastic processes (e.g., dispersal limitation) were dominant, and deterministic assembly (e.g., homogeneous selection) had a tendency to increase from LF to HF. There is a slight difference in the trend of the proportion of the stochastic process in the two algorithms which is probably because the effect size of the influence of dispersal limitation is different for abundant and rare taxa (Weiss et al., 2016) and further research is needed. However, the results of the null model and neutral model analyses support the notion that stochastic processes dominate the assembly of bacteria communities in tobacco soil ecosystems.

## 5. Conclusion

The study revealed that as the comprehensive soil fertility index increases, microbial diversity tends to increase, with community differences primarily observed between HF and LF soils. HF soils exhibits a higher proportion of Proteobacteria, while Acidobacteria and Actinobacteria are more dominant in MF and LF soils. Microbial communities in tobacco planting soils with varying comprehensive fertilities demonstrate a stochastic assembly process, where the dissimilarities in microbial communities between MF and LF soils increase as environmental distance increases; however, no significant differences are observed in HF soils microbial communities. The properties of pH, SOM, and WSCa may serve as key factors influencing the composition of microbial communities. Additionally, stronger interspecies cooperation is observed among microbial communities in HF soils. These research findings provide theoretical references for studying environmental factors and microbial communities in tobacco planting soils while further elucidating the characteristics of soil microbial communities under conditions of comprehensive soil fertility and their influencing factors, thereby providing a reference basis for evaluating soil health.

## Data availability statement

The datasets presented in this study can be found in online repositories. The names of the repository/repositories and accession number(s) can be found at: https://www.ncbi.nlm.nih.gov/, PRJNA976908.

## Author contributions

CY and YZ contributed to the study’s conception and design. Material preparation were performed by ZZ, YC, and GY. Data collection and analysis were performed by YX, TL, and HD. The first draft of the manuscript was written by YX and TL. All authors contributed to the article and approved the submitted version.

## Funding

This work was funded by the Key Project of China National Tobacco Corporation (11202102037). The funder has no involvement in the study design, analysis, interpretation of data, writing of the article or the decision to submit it for publication.

## Conflict of interest

TL, HD, ZZ, and YZ were employed by Zhengzhou Tobacco Research Institute of CNTC. YC was employed by Liangshan Branch of Sichuan Tobacco Company. GY was employed by China Tobacco Henan Industrial Co., Ltd.

The remaining authors declare that the research was conducted in the absence of any commercial or financial relationships that could be construed as a potential conflict of interest.

## Publisher’s note

All claims expressed in this article are solely those of the authors and do not necessarily represent those of their affiliated organizations, or those of the publisher, the editors and the reviewers. Any product that may be evaluated in this article, or claim that may be made by its manufacturer, is not guaranteed or endorsed by the publisher.
