# Supplementary material for: Characteristics of soil microbial communities in farmland with different comprehensive fertility levels in the Panxi area, Sichuan, China
Source: Front Microbiol. 2023 Sep 14;14:1237409. doi: 10.3389/fmicb.2023.1237409 (PMC10539910; doi:10.3389/fmicb.2023.1237409)
Supplement: Supplementary file 1 [file Table_1.pdf]

Characteristics of soil microbial communities in farmland with different comprehensive fertility levels in the Panxi area, Sichuan, China

Yadong Xu[1,3†], Taibo Liang[2†]，Huaxin Dai[2]，Zhen Zhai[2], Yulan Chen[4], Guangting Yin[5], Yanling Zhang[2*], Caipeng Yue[1,3**]

[1] School of Agricultural Sciences, Zhengzhou University, Zhengzhou, Henan, 450001, China.

[2] Zhengzhou Tobacco Research Institute of CNTC, Zhengzhou, Henan, 450001, China.

[3] Henan Funiu Mountain Biological and Ecological Environment Observatory Research Project, Zhengzhou, Henan, 450001, China.

[4] Liangshan Branch of Sichun Tobacco Company, Xicang, Sichuan, 615000, China.

[5] China Tobacco Henan Industrlal Co., Ltd, Zhengzhou, Henan, 450001, China.

[†] Yadong Xu and Taibo Liang contributed equally to this work.

* Corresponding author: Yanling Zhang, Zhengzhou Tobacco Research Institute of CNTC, Zhengzhou, Henan, 450001, China. Email: zhangyanling@ztri.com.cn;

** Corresponding author: Caipeng Yue, School of Agricultural Sciences, Zhengzhou University, Zhengzhou, Henan, 450001, China. yuecaipeng@zzu.edu.cn.

Supplementary Table S1 Geographic information of the sampling point.

	Latitude (°)	Longitude (°)	Altitude (m)
LF1	26.58N	102.35E	1761.00
LF2	26.57N	102.35E	1815.00
LF3	26.61N	102.37E	1852.00
LF4	26.57N	102.38E	1779.00
LF5	26.57N	102.41E	1709.00
LF6	26.56N	102.41E	1775.00
LF7	26.56N	102.43E	1773.00
LF8	26.5N	102.63E	1894.00
LF9	26.47N	102.63E	1972.00
LF10	26.46N	102.64E	2023.00
LF11	26.42N	102.65E	2098.00
LF12	26.43N	102.63E	1951.00
LF13	26.59N	102.50E	1850.00
LF14	26.59N	102.50E	1868.00
LF15	26.64N	102.33E	1800.12
LF16	26.69N	102.37E	1881.70
LF17	26.34N	102.09E	1759.04
LF18	26.33N	102.10E	1751.03
LF19	26.82N	102.28E	2074.98
LF20	26.82N	102.27E	2082.89

LF21	28.12N	105.12E	447.50
LF22	28.24N	104.99E	1040.21
LF23	28.23N	104.99E	1010.01
LF24	27.87N	104.61E	1125.09
LF25	27.89N	104.63E	1034.40
LF26	27.87N	104.58E	1091.56
LF27	27.88N	104.56E	1140.27
LF28	27.92N	104.50E	1172.96
LF29	27.92N	105.59E	1023.05
LF30	27.92N	105.66E	1274.00
LF31	28.54N	102.47E	1798.00
LF32	28.64N	102.51E	1630.00
LF33	28.72N	102.53E	1637.00
LF34	28.72N	102.51E	1717.00
MF1	26.61N	102.37E	1900.00
MF2	26.57N	102.38E	1779.00
MF3	26.56N	102.41E	1770.00
MF4	26.56N	102.43E	1774.00
MF5	26.49N	102.62E	1867.00
MF6	26.46N	102.64E	2021.00
MF7	26.47N	102.63E	1963.00
MF8	26.43N	102.63E	1960.00

MF9	26.43N	102.63E	2150.00
MF10	26.47N	102.64E	2110.00
MF11	26.43N	102.63E	1980.00
MF12	26.65N	102.44E	1969.00
MF13	26.65N	102.44E	2135.00
MF14	26.65N	102.44E	2130.00
MF15	26.67N	102.41E	2037.00
MF16	26.59N	102.5E	1840.00
MF17	26.59N	102.48E	1944.00
MF18	26.59N	102.5E	1904.00
MF19	26.67N	102.62E	2226.00
MF20	26.69N	102.62E	2318.00
MF21	26.74N	102.6E	2166.00
MF22	26.64N	102.27E	1755.35
MF23	26.64N	102.27E	1783.82
MF24	26.75N	102.47E	1879.91
MF25	26.75N	102.47E	1889.71
MF26	26.68N	102.36E	1866.26
MF27	26.38N	102.16E	1759.08
MF28	26.38N	102.16E	1761.90
MF29	26.38N	102.15E	1784.53
MF30	26.31N	102.19E	1778.06

MF31	26.31N	102.19E	1829.74
MF32	26.31N	102.19E	1831.24
MF33	26.38N	101.99E	1800.24
MF34	26.29N	102.01E	1780.00
MF35	26.75N	102.25E	1942.62
MF36	26.75N	102.26E	1887.21
MF37	26.82N	102.28E	2076.41
MF38	26.82N	102.27E	2083.64
MF39	26.82N	102.27E	2087.00
MF40	26.83N	102.28E	2062.10
MF41	26.82N	102.28E	2060.90
MF42	28.12N	105.12E	448.40
MF43	28.13N	105.08E	864.14
MF44	28.19N	105.07E	1153.63
MF45	28.23N	104.99E	1030.57
MF46	28.19N	105E	1178.86
MF47	27.97N	104.85E	914.03
MF48	27.97N	104.84E	947.46
MF49	27.98N	104.88E	679.73
MF50	27.94N	104.78E	767.18
MF51	27.94N	104.51E	1105.87
MF52	28.56N	102.48E	1739.00

MF53	28.56N	102.5E	1705.00
MF54	28.53N	102.5E	1688.00
MF55	28.6N	102.49E	1680.00
MF56	28.61N	102.52E	1640.00
HF1	26.57N	102.37E	1788.00
HF2	26.56N	102.41E	1773.00
HF3	26.47N	102.63E	1945.00
HF4	26.43N	102.63E	1963.00
HF5	26.45N	102.63E	1952.00
HF6	26.43N	102.63E	2150.00
HF7	26.65N	102.44E	1974.00
HF8	26.66N	102.42E	2185.00
HF9	26.66N	102.42E	2185.00
HF10	26.67N	102.41E	2037.00
HF11	26.59N	102.50E	1850.00
HF12	26.61N	102.52E	1970.00
HF13	26.61N	102.52E	1972.00
HF14	26.68N	102.64E	2211.00
HF15	26.71N	102.62E	2539.00
HF16	26.72N	102.60E	3434.00
HF17	26.73N	102.61E	2328.00
HF18	26.75N	102.47E	1885.15

HF19	26.38N	101.99E	1799.59
HF20	26.38N	101.99E	1799.42
HF21	26.35N	102.08E	1824.41
HF22	26.75N	102.25E	1933.78
HF23	26.75N	102.26E	1887.04
HF24	26.82N	102.28E	2077.86
HF25	26.82N	102.27E	2076.60
HF26	26.82N	102.28E	2073.73
HF27	26.81N	102.25E	2296.68
HF28	26.81N	102.25E	2293.61
HF29	27.93N	104.57E	1166.70
HF30	27.91N	104.57E	1121.84
HF31	28.57N	102.49E	1700.00
HF32	28.6N	102.49E	1680.00
HF33	28.61N	102.50E	1695.00
